# Community-Level Disadvantage of Adults With Firearm- vs Motor Vehicle–Related Injuries

**DOI:** 10.1001/jamanetworkopen.2024.19844

**Published:** 2024-07-05

**Authors:** Lauren L. Agoubi, Samantha Banks, Ashley B. Hink, Deborah Kuhls, Shelbie D. Kirkendoll, Alex Winchester, Christopher Hoeft, Bhavin Patel, Avery Nathens

**Affiliations:** 1 Harborview Injury Prevention and Research Center and the Department of Surgery, University of Washington, Seattle; 2 Firearm Injury and Policy Research Program, University of Washington, Seattle; 3 Department of Surgery, Medical University of South Carolina, Charleston; 4 Department of Surgery, Kirk Kerkorian School of Medicine at University of Nevada, Las Vegas; 5 Department of Surgery, Northwestern Feinberg School of Medicine, Chicago, Illinois; 6 American College of Surgeons; 7 Department of Surgery, Sunnybrook Health Sciences Center, University of Toronto, Toronto, Ontario, Canada

## Abstract

**Importance:**

Motor vehicle crash (MVC) and firearm injuries are 2 of the top 3 mechanisms of adult injury-related deaths in the US.

**Objective:**

To understand the differing associations between community-level disadvantage and firearm vs MVC injuries to inform mechanism-specific prevention strategies and appropriate postdischarge resource allocation.

**Design, Setting, and Participants:**

This multicenter cross-sectional study analyzed prospectively collected data from the American College of Surgeons (ACS) Firearm Study. Included patients were treated either for firearm injury between March 1, 2021, and February 28, 2022, or for MVC-related injuries between January 1 and December 31, 2021, at 1 of 128 participating ACS trauma centers.

**Exposures:**

Community distress.

**Main outcome and Measure:**

Odds of presenting with a firearm as compared with MVC injury based on levels of community distress, as measured by the Distressed Communities Index (DCI) and categorized in quintiles.

**Results:**

A total of 62 981 patients were included (mean [SD] age, 42.9 [17.7] years; 42 388 male [67.3%]; 17 737 Black [28.2%], 9052 Hispanic [14.4%], 36 425 White [57.8%]) from 104 trauma centers. By type, there were 53 474 patients treated for MVC injuries and 9507 treated for firearm injuries. Patients with firearm injuries were younger (median [IQR] age, 31.0 [24.0-40.0] years vs 41.0 [29.0-58.0] years); more likely to be male (7892 of 9507 [83.0%] vs 34 496 of 53 474 [64.5%]), identified as Black (5486 of 9507 [57.7%] vs 12 251 of 53 474 [22.9%]), and Medicaid insured or uninsured (6819 of 9507 [71.7%] vs 21 310 of 53 474 [39.9%]); and had a higher DCI score (median [IQR] score, 74.0 [53.2-94.8] vs 58.0 [33.0-83.0]) than MVC injured patients. Among admitted patients, the odds of presenting with a firearm injury compared with MVC injury were 1.50 (95% CI, 1.35-1.66) times higher for patients living in the most distressed vs least distressed ZIP codes. After controlling for age, sex, race, ethnicity, and payer type, the DCI components associated with the highest adjusted odds of presenting with a firearm injury were a high housing vacancy rate (OR, 1.11; 95% CI, 1.04-1.19) and high poverty rate (OR, 1.17; 95% CI, 1.10-1.24). Among patients sustaining firearm injuries patients, 4333 (54.3%) received no referrals for postdischarge rehabilitation, home health, or psychosocial services.

**Conclusions and Relevance:**

In this cross-sectional study of adults with firearm- and motor vehicle–related injuries, we found that patients from highly distressed communities had higher odds of presenting to a trauma center with a firearm injury as opposed to an MVC injury. With two-thirds of firearm injury survivors treated at trauma centers being discharged without psychosocial services, community-level measures of disadvantage may be useful for allocating postdischarge care resources to patients with the greatest need.

## Key Points

**Question:**

How do measures of community distress differ for adult patients with motor vehicle– vs firearm-related injuries and what services do patients injured by firearms receive at discharge?

**Findings:**

In this cross-sectional study of 62 981 adults seeking trauma center care for motor vehicle and firearm injuries, the odds of presenting to the emergency department with a firearm injury vs motor vehicle crash–related injury were 50% higher for patients living in the most vs least distressed zip codes. Among patients with firearm injuries, 54% received no referral for postdischarge services.

**Meaning:**

These results suggest that patients injured by firearms come from communities with higher economic disadvantage than patients injured in motor vehicle crashes, and as such may require more postdischarge services to recover.

## Teaser

This cross-sectional study of US adults compares rates of injuries related to firearm use vs motor vehicles crashes relative to community-level social determinants of health.

## Introduction

Among adults, injuries due to motor vehicle crashes (MVCs) and firearms remain 2 of the top 3 causes of injury-related deaths in the US.^1,2^ MVC-related deaths declined substantially in the last half of the 20th century, in part due to a multifaceted public health approach focused- less on the individual and more on their context, bolstered by robust injury surveillance systems.^3,4^ By contrast, firearm deaths have continued to rise, with firearm-injury prevention research historically handicapped by federal funding restrictions and limited person-level data.^5,6^

Community-level determinants of health have been associated with vulnerability to injuries.^7,8,9^ The development of community-level indices enabled public health researchers to examine the aggregate impact of upstream social determinants of health (SDOH) that affect a person’s risk of injury. Using these indices, prior work has demonstrated that patients experiencing community-level disadvantage are more vulnerable to injury, and sustain more severe injuries.^9,10^ While the association between community-level determinants of health and injury mechanism has been compared among pediatric patients,^11^ this has not been examined at the national level in the adult population. Additionally, prior evaluations of the associations between community-level socioeconomics and firearm injury compared with other injury mechanisms have reported geographically limited experiences with relatively small sample sizes.^10,12,13^ Understanding how community-level disadvantage is associated with MVC and firearm injuries and how these associations differ may inform policymaking and resource allocation for interventions and supportive recovery services.

The absence of comprehensive firearm injury data has affected researchers’ ability to take a public health approach in responding to the rise in firearm injuries and deaths.^14^ Limitations of existing data sources include a reliance on hospital billing data, which are not granular and are devoid of critical information regarding context of injury, absence of contemporaneous data, and a focus on fatal firearm injuries.^6^ Given this need for expanded firearm injury surveillance, the American College of Surgeons (ACS) Committee on Trauma (COT) leveraged the Trauma Quality Improvement Program (TQIP) infrastructure to collect additional data on patients injured with firearms presenting to participating trauma centers, including data focused on patient history, injury context, and postinjury care.

Using data from the ACS COT Firearm Study,^15^ we sought to describe patient and injury characteristics of patients with firearm injuries compared with patients injured in MVCs, and to contrast associations with community distress by mechanisms of injury.^16^ We hypothesized that community distress would differ across the 2 populations, but that patients from the most distressed communities would be most vulnerable to both mechanisms of injury. Additionally, we aimed to assess the provision of postdischarge services for patients injured by firearms.

## Methods

### Study Design

Our aim was to compare risk factors for firearm vs MVC injury, with a focus on SDOH measured by levels of community distress. We used data derived from a prospective cohort study of patients injured with firearms across the US and data from a population of patients cared for at the same trauma centers with MVC injuries.

### Data Sources and Study Population

One hundred sixty-five trauma centers volunteered to participate in the ACS COT Firearm Study,^15^ with 128 centers ultimately contributing data. A comparison of participating vs nonparticipating TQIP centers has been previously described, with the primary difference being overrepresentation of level I and II centers in the study.^16^ Adult patients aged 19 years or older who presented alive to a participating trauma center were included. All patients who sustained a firearm injury, regardless of intent, between March 1, 2021, and February 28, 2022, were included. Patients with MVC-related injuries between January 1 and December 31, 2021, were included.^16^ Although the ACS Firearm Study collected data on patients discharged alive from the emergency department (ED), the National Trauma Data Standard (NTDS) does not routinely collect data on patients discharged alive from the ED. For the purpose of consistency between the samples for patients injured by firearm and MVCs, patients discharged alive from the ED were excluded. Patients with an MVC-related injury (occupant, motorcyclists, pedal cyclist, pedestrian, and unspecified) were identified using the TQIP mechanisms of injury and cross-checked against *International Statistical Classification of Diseases and Related Health Problems, Tenth Revision (ICD-10)* external cause of injury codes. Only facilities that submitted data on at least 1 patient aged 19 years or older of each injury type were included in analyses.

As part of the ACS Firearm Study, patient-level demographic, injury, hospital, and discharge characteristics were captured through the TQIP infrastructure using the 2021 NTDS data dictionary.^17^ All additional data elements regarding patient level risks, injury circumstances, and supportive discharge services were abstracted from the medical record.^18^ Some data elements collected specifically for firearm injuries were not collected on MVC injuries.

This study was approved by Advarr Center for Institutional Review Board Intelligence, and was exempt from informed consent requirements as all data were deidentified. The Reporting of Studies Conducted Using Observational Routinely-Collected Data (RECORD) reporting guidelines for cross-sectional studies were followed in the writing of this manuscript. Methods specific to the ACS Firearm study protocol and data acquisition have been described in detail elsewhere.^16^

### Study Variables

The outcome of interest in this study was odds of a firearm vs motor vehicle injury. To determine the association between community levels of distress with firearm as compared with MVC injuries, we used the Distressed Community Index (DCI) as a measure of community-level socioeconomic disadvantage. The DCI was created by the Economic Innovation Group to measure economic well-being of US communities using data derived from the US Census Bureau’s Business Patterns and American Community Survey 5-year estimates for 2016-2020.^19^ The DCI provides a composite measure ranging from 0 to 100, ranging from least to most distressed and integrating percentage of adults with a high school diploma, housing vacancy rate, percentage of adults not working, poverty rate, median income ratio, percentage change in employment, and percentage change in number of business establishments over the time period. For the purposes of the DCI, median income ratio is defined as “the median household income as a proportion of metro area median household income.”^19^ The zip codes used in this study refer to where the patient resided.^19^ The DCI is not available for zip codes with fewer than 500 residents. For our analysis, we grouped the cohort into DCI quintiles.

Additional covariates were identified a priori and included age, sex, race, ethnicity, and payer type. Race was included as a proxy for racism both in social drivers of injury and in systematic racism in health care. NTDS race categories were based on the 2010 US Census Bureau and self-selected by patients or a family member. Race categories included American Indian, Asian, Black, other, Pacific Islander, White, more than 1 listed race, and missing. Other race is not defined in the NTDS. Ethnicity categories included Hispanic or Latino, non-Hispanic or Latino, and missing.

Data on postdischarge service referral was collected as a part of the expanded data collection for patients injured by firearms and included rehabilitation, home health, or psychosocial services.^18^ Rehabilitation included inpatient rehabilitation, outpatient physical therapy, occupational therapy, speech therapy, or rehabilitation medicine. Home health needs included nursing, wound care, infusion therapy, and rehabilitation therapies. Psychosocial ancillary services included social work or case management, child protective services, psychology, psychiatry, violence intervention programs, intimate partner services, and housing services. Services were not mutually exclusive.

### Statistical Analysis

Descriptive statistics were calculated and reported by injury type for demographic, clinical, and zip-code based variables. Proportions of patients receiving postdischarge services were calculated and reported among the firearm injury group.

We created 2 binomial generalized linear mixed-effects models with the dependent variable being firearm injury. Thus, model outputs represent the associations between the risk factors at the individual and community level in patients injured by firearms, with the reference being patients with MVC-related injuries. First, a binomial generalized linear mixed-effects model was fit to estimate the association between DCI quintile and injury type, controlling for age group, sex, race, ethnicity, and payer type. Clustering at the facility level was accounted for with a random intercept. Then, a separate binomial generalized linear mixed-effects model was fit to estimate the associations between each component of the DCI score (listed previously). The DCI components were included in the model as continuous variables, scaled so that the model results represented 10-unit differences. Age group, sex, race, ethnicity, and payer type were controlled for odds ratios, with 95% confidence intervals and *P* values at the .05 α level reported for both models. All data cleaning and analysis was performed using Rstudio version 2022.07.2 (R Project for Statistical Computing).

## Results

### Demographics and Patient Characteristics

After excluding patients younger than 19 years and trauma centers that did not treat at least 1 patient of each injury type, firearm injury data were included from 104 trauma centers. Seven hundred ninety unique zip codes were excluded based on the described DCI methodology, corresponding to 2078 patients. A total of 62 981 patients met our inclusion criteria (median [IQR] age, 39 [28-56] years; 42 388 male [67.3%]; 17 737 Black [28.2%], 9052 Hispanic [14.4%], 36 425 White [57.8%]). Of these, 53 474 (84.9%) patients were treated for MVC-related injuries, and 9507 (15.1%) were treated for firearm-related injuries (Table 1; eTable 1 in Supplement 1). Among patients with firearm injuries, 6753 (71.0%) were injured by assault, 867 (9.1%) unintentionally, 643 (6.8%) by self-intent, and 131 (1.4%) by law enforcement. Compared with patients injured in MVCs, patients injured by firearms were younger (median [IQR] age, 31.0 [24.0-40.0] years vs 41.0 [29.0-58.0] years) and more likely to be male (7892 of 9507 [83.0%] vs 34 496 of 53 474 [64.5%]), identified as Black (5486 of 9507 [57.7%] vs 12 251 of 53 474 [22.9%]), and Medicaid insured or uninsured (6819 of 9507 [71.7%] vs 21 310 of 53 474 [39.9%]). The regional distribution of facilities was similar in both groups.

**Table 1.** Patient Demographics Comparing Adult Motor Vehicle Injury With Patients With Firearm Injuries^a^

Characteristics	Patients, No. (%)		
	Firearm injury (n = 9507)	Motor vehicle collision injury (n = 53 474)	Overall (N = 62 981)
Age, y			
Mean (SD)	33.9 (12.9)	44.5 (18.0)	42.9 (17.7)
Median (IQR)	31.0 (24-40)	41 (29-58)	39 (28-56)
19-30	4704 (49.5)	15 509 (29.0)	20 213 (32.1)
31-40	2567 (27.0)	10 391 (19.4)	12 958 (20.6)
41-50	1193 (12.5)	7854 (14.7)	9047 (14.4)
51-64	736 (7.7)	10 926 (20.4)	11 662 (18.5)
≥65	307 (3.2)	8794 (16.4)	9101 (14.5)
Sex			
Female	1196 (12.6)	18 150 (33.9)	19 346 (30.7)
Male	7892 (83.0)	34 496 (64.5)	42 388 (67.3)
Nonbinary	0	8 (<0.1)	8 (<0.1)
Missing	419 (4.4)	820 (1.5)	1239 (2.0)
Race			
American Indian	57 (0.6)	363 (0.7)	420 (0.7)
Asian	83 (0.9)	986 (1.8)	1069 (1.7)
Black	5486 (57.7)	12 251 (22.9)	17 737 (28.2)
Pacific Islander	40 (0.4)	228 (0.4)	268 (0.4)
White	2762 (29.1)	33 663 (63.0)	36 425 (57.8)
Other^b^	720 (7.6)	4644 (8.7)	5364 (8.5)
>1 Race	21 (0.2)	115 (0.2)	136 (0.2)
Missing	338 (3.6)	1224 (2.3)	1562 (2.5)
Ethnicity			
Hispanic or Latino	1308 (13.8)	7744 (14.5)	9052 (14.4)
Not Hispanic or Latino	7627 (80.2)	43 888 (82.1)	51 515 (81.8)
Missing	572 (6.0)	1842 (3.4)	2414 (3.8)
Intent			
Assault	6753 (71.0)	NA	NA
Law enforcement	131 (1.4)	NA	NA
Self-inflicted	643 (6.8)	NA	NA
Unintentional	867 (9.1)	NA	NA
Missing	1113 (11.7)	NA	NA
Primary method of payment			
Medicaid/other government	4147 (43.6)	12 689 (23.7)	16 836 (26.7)
Medicare	426 (4.5)	6368 (11.9)	6794 (10.8)
Private/commercial insurance	1800 (18.9)	23 835 (44.6)	25 635 (40.7)
Uninsured/other	2672 (28.1)	8621 (16.1)	11 293 (17.9)
Missing	462 (4.9)	1961 (3.7)	2423 (3.8)
Facility region			
Midwest	2829 (29.8)	15 308 (28.6)	18 137 (28.8)
Northeast	1374 (14.5)	6602 (12.3)	7976 (12.7)
South	3294 (34.6)	21 309 (39.8)	24 603 (39.1)
West	2010 (21.1)	10 255 (19.2)	12 265 (19.5)

Abbreviation: NA, not applicable.

^a^ Patients discharged alive from the emergency department were excluded.

^b^ Other race is not defined in the National Trauma Data Standard and is self-selected by a patient or family member.

### Clinical Characteristics and Discharge Services

Mortality was the result for 4694 patients (7.5%) in the overall cohort, with a greater proportion of deaths in the firearm-injured group (1533 of 9507 patients [16.1%] vs 3161 of 53 474 patients [5.9%]) (Table 2; eTable 2 in Supplement 1). Patients with MVC injuries were more frequently transported by air than patients with firearm injuries, whereas firearm-injured cohort was more often transported by police or personal vehicle. Compared with patients with MVC injuries, a larger proportion of patients with firearm injuries died in the ED (892 of 9507 [9.4%] vs 1101 of 53 474 [2.1%]) or had an ED disposition of the operating room (3779 of 9507 [39.7%] vs 8145 of 53 474 [15.2%]). Median ICU and overall hospital LOS were similar between groups. Patients sustaining MVCs were more frequently discharged to a skilled nursing facility or a long-term care facility than patients injured with firearms.

**Table 2.** Clinical Characteristics by Injury Type

Characteristics	Patients, No. (%)		
	Firearm injury (n = 9507)	Motor vehicle collision injury (n = 53 474)	Overall (n = 62 981)
Mortality			
Deceased	1533 (16.1)	3161 (5.9)	4694 (7.5)
Survived	7974 (83.9)	50 313 (94.1)	58 287 (92.5)
Transport mode			
Air	923 (9.7)	8006 (15.0)	8929 (14.2)
Ground ambulance	7090 (74.6)	43 154 (80.7)	50 244 (79.8)
Other	4 (<0.1)	18 (<0.1)	22 (<0.1)
Police	219 (2.3)	71 (0.1)	290 (0.5)
Private, public vehicle, or walk-in	1251 (13.2)	2099 (3.9)	3350 (5.3)
Missing	20 (0.2)	126 (0.2)	146 (0.2)
ED discharge disposition			
Died in ED	892 (9.4)	1101 (2.1)	1993 (3.2)
Floor/observation unit	2925 (30.8)	25 124 (47.0)	28 049 (44.5)
ICU/SDU	1807 (19.0)	18 293 (34.2)	20 100 (31.9)
OR	3779 (39.7)	8145 (15.2)	11 924 (18.9)
Missing	104 (1.1)	811 (1.5)	915 (1.5)
Total length of stay			
Mean (SD), d	7.4 (10.1)	8.3 (11.2)	8.2 (11.1)
Median (range), d	4.0 (1.0-169.0)	5.0 (1.0-368.0)	5.0 (1.0-368.0)
Missing	200 (2.1)	109 (0.2)	309 (0.5)
ICU length of stay			
Mean (SD)	6.3 (8.5)	6.9 (8.6)	6.8 (8.6)
Median (range)	3.0 (1.0-142.0)	4.0 (1.0-138.0)	4.0 (1.0-142.0)
Hospital discharge disposition among admitted, No.	8615	52 373	60 988
Died	641 (7.4)	2060 (3.9)	2701 (4.4)
Home	6317 (73.3)	37 195 (71.0)	43 512 (71.3)
Other	677 (7.9)	1689 (3.2)	2366 (3.9)
SNF or other long-term care	903 (10.5)	11 035 (21.1)	11 938 (19.6)
Transferred to other acute care facility	46 (0.5)	394 (0.8)	440 (0.7)
Missing	31 (0.4)	0	31 (<0.1)
Alcohol screen result			
Negative	4415 (46.4)	24 690 (46.2)	29 105 (46.2)
Positive	2108 (22.2)	11 451 (21.4)	13 559 (21.5)
Missing/not tested	2984 (31.4)	17 333 (32.4)	20 317 (32.3)

Abbreviations: ED, emergency department; ICU, intensive care unit; OR, operating room; SDU, step down unit; SNF, skilled nursing facility.

Rehabilitation and postdischarge service variables were available solely for patients with firearm injuries and are shown in Table 3 stratified by injury severity score (ISS) (eTable 3 in Supplement 1). Of these individuals, 54.3% were not referred for postdischarge rehabilitation, home health, or psychosocial services. Patients with higher ISS were more likely to be referred for postdischarge services. Of those with services, psychosocial ancillary services were the most common referral services, with 2154 patients (27%) referred for some form of psychosocial care. The 2 most common psychosocial services were hospital- or community-based violence intervention programs (1072 [13.4%]) and social work or case management (829 [10.4%]).

**Table 3.** Postdischarge Services Among Surviving Adult Patients With Firearm Injuries Stratified by Injury Severity Score (ISS)^a^

Service	ISS, No. (%)			
	<16 (n = 5946)	16-24 (n = 1124)	≥25 (n = 890)	Overall (N = 7974)
Rehabilitation/postdischarge needs				
Any	1006 (16.9)	313 (27.8)	422 (47.4)	1743 (21.9)
Inpatient subacute rehabilitation	302 (5.1)	155 (13.8)	308 (34.6)	766 (9.6)
Outpatient physical therapy	554 (9.3)	121 (10.8)	98 (11.0)	774 (9.7)
Outpatient occupational therapy	283 (4.8)	80 (7.1)	63 (7.1)	426 (5.3)
Outpatient speech therapy	27 (0.5)	15 (1.3)	15 (1.7)	57 (0.7)
Outpatient rehabilitation medicine	72 (1.2)	17 (1.5)	30 (3.4)	119 (1.5)
Missing	365 (6.1)	75 (6.7)	54 (6.1)	495 (6.2)
Home health needs				
Any	859 (14.4)	231 (20.6)	218 (24.5)	1311 (16.4)
Nursing	340 (5.7)	116 (10.3)	106 (11.9)	562 (7.0)
Wound care	406 (6.8)	92 (8.2)	72 (8.1)	573 (7.2)
Infusion therapy	8 (0.1)	7 (0.6)	6 (0.7)	21 (0.3)
Rehabilitation therapies	350 (5.9)	93 (8.3)	104 (11.7)	547 (6.9)
Missing	397 (6.7)	116 (10.3)	126 (14.2)	640 (8.0)
Psychosocial ancillary services				
Any	1443 (24.3)	374 (33.3)	335 (37.6)	2154 (27.0)
Social work/case manager	574 (9.7)	138 (12.3)	116 (13.0)	829 (10.4)
Child protective services	11 (0.2)	2 (0.2)	3 (0.3)	16 (0.2)
Psychologist	172 (2.9)	42 (3.7)	58 (6.5)	272 (3.4)
Psychiatry	233 (3.9)	89 (7.9)	66 (7.4)	388 (4.9)
Hospital/community-based violence intervention programs	728 (12.2)	165 (14.7)	178 (20.0)	1072 (13.4)
Intimate partner violence services	21 (0.4)	7 (0.6)	4 (0.4)	32 (0.4)
Housing services (shelter, transitional housing)	103 (1.7)	26 (2.3)	15 (1.7)	144 (1.8)
Missing	380 (6.4)	92 (8.2)	86 (9.7)	560 (7.0)

^a^ Percentages for specific services are calculated among all surviving patients with firearm injuries and are not mutually exclusive.

### Distressed Communities Index

Overall, the cohorts had a median (IQR) DCI score of 66.6 (35.6-85.6) (Table 4; eTable 4 in Supplement 1). Patients in the firearm injury group had a higher median (IQR) DCI score than the MVC group (74.0 [53.2-94.8] vs 58.0 [33.0-83.0]). For both groups, the proportion of injured patients was underrepresented in the least distressed quintiles and overrepresented in the most distressed quintiles. In comparing each component of the DCI between patients with firearm- vs MVC-related injuries, patients injured with firearms were from communities with higher distress for each component, with the largest differences in the poverty rate and median income ratios.

**Table 4.** Distressed Communities Index (DCI) of Patient Residential Zip Codes by Injury Type

Characteristics	Patients, No. (%)		
	Firearm injury (n = 9507)	Motor vehicle collision injury (n = 53 474)	Overall (N = 62 981)
Mean (SD) score	66.1 (27.1)	54.8 (29.0)	56.5 (29.0)
Median (IQR) score	74.0 (53.2-94.8)	58.0 (33.0-83.0)	66.6 (35.6-85.6)
Quintiles			
1 (Least distressed)	788 (8.3)	8727 (16.3)	9515 (15.1)
2	1044 (11.0)	8552 (16.0)	9596 (15.2)
3	1358 (14.3)	9555 (17.9)	10913 (17.3)
4	2190 (23.0)	11549 (21.6)	13739 (21.8)
5 (Most distressed)	3746 (39.4)	13394 (25.0)	17140 (27.2)
Missing	381 (4.0)	1697 (3.2)	2078 (3.3)
DCI subcomponents			
No high school diploma, median (range), %	14.1 (0 to 61.4)	11.7 (0 to 72.0)	12.1 (0 to 72.0)
Poverty rate, median (range), %	18.8 (0 to 63.8)	14.1 (0 to 81.2)	14.7 (0 to 81.2)
Adults not working, median (range), %	24.4 (0 to 96.0)	21.9 (0 to 97.2)	22.3 (0 to 97.2)
Housing vacancy rate, median (range), %	9.1 (0 to 53.4)	7.5 (0 to 55.3)	7.7 (0 to 55.3)
Median income ratio, median (range)^a^	73.8 (21.7 to 264.0)	87.8 (4.1 to 317.0)	86.2 (4.1 to 317.0)
Change in employment, median (range), %	3.5 (−76.2 to 480.0)	4.2 (−89.1 to 2610.0)	4.1 (−89.1 to 2610.0)
Change in establishments, median (range), %	1.9 (−53.6 to 133.0)	2.5 (−82.1 to 400.0)	2.4 (−82.1 to 400.0)

Abbreviation: DCI, Distressed Communities Index.

^a^ Median income ratio includes median household income as a proportion of metropolitan area median household income.

On adjusted multivariable regression analysis, the odds of presenting to the ED with a firearm injury rather than an MVC-related injury were 1.50 (95% CI, 1.35-1.66) times higher for patients living in zip codes in the most distressed quintile compared with the least distressed quintile and 1.26 (95% CI, 1.13-1.40) times higher for patients from the second most distressed quintile compared with the least distressed quintile (Table 5; eTable 5 in Supplement 1). Patients who were identified as Black, male, Medicaid insured, or uninsured had over 3 times higher adjusted odds of presenting with a firearm vs MVC injury. The components of the DCI associated with the highest adjusted odds of presenting with a firearm injury were residence in a community with a high housing vacancy rate (OR, 1.11; 95% CI, 1.04-1.19) and high poverty rate (OR, 1.17; 95% CI, 1.10-1.24).

**Table 5.** Adjusted Odds Ratios (OR) of Firearm Injury vs Motor Vehicle Crash (MVC)–Related Injury^a^

Characteristic	OR (95% CI)
Age group, y	
19-30	1 [Reference]
31-40	0.81 (0.76-0.86)
41-50	0.54 (0.50-0.59)
51-64	0.27 (0.24-0.30)
≥65	0.20 (0.17-0.23)
Sex	
Male	1 [Reference]
Female	0.31 (0.29-0.33)
Race	
White	1 [Reference]
American Indian	1.37 (0.99-1.90)
Asian	1.24 (0.95-1.61)
Black	3.93 (3.66-4.21)
Other^b^	1.25 (1.10-1.41)
Pacific Islander	2.44 (1.60-3.70)
>1 Race	1.82 (1.06-3.13)
Ethnicity	
Not Hispanic/Latino	1 [Reference]
Hispanic/Latino	1.21 (1.09-1.34)
Payer type	
Private/commercial insurance	1 [Reference]
Medicare	2.39 (2.07-2.77)
Medicaid/other government	3.64 (3.38-3.91)
Self-pay, not billed, or other	3.14 (2.89-3.41)
DCI quintile	
1 (Least distressed)	1 [Reference]
2	1.08 (0.96-1.21)
3	1.07 (0.96-1.19)
4	1.26 (1.13-1.40)
5 (Most distressed)	1.50 (1.35-1.66)
DCI subcomponent analysis	
No high school diploma	0.98 (0.93-1.02)
Poverty	1.17 (1.10-1.24)
Adults not working	0.97 (0.92-1.02)
Housing vacancy rate	1.11 (1.04-1.19)
Median income ratio	0.99 (0.98-1.01)
Change in employment	1.00 (0.98-1.01)
Change in establishments	1.00 (0.97-1.04)

Abbreviation: DCI, Distressed Communities Index.

^a^ Clustering at the facility level was controlled for with a random intercept. ORs presented for the DCI subcomponent analysis control for sociodemographic characteristics.

^b^ Other race is not defined in the National Trauma Data Standard and is self-selected by a patient or family member.

## Discussion

In this large, multicenter study of adult patients admitted to trauma centers across the US, we found that patients from the most distressed communities had 35% to 66% greater odds of presenting with a firearm injury compared with an MVC injury. Furthermore, over half of all firearm injury survivors were not referred for rehabilitation or postdischarge services, and only 27% of these patients receive a referral for any form of psychosocial care, such as violence intervention services, social work consultation, or case management. Taken together, these findings suggest that patients sustaining firearm injuries not only come from highly vulnerable areas, but that the majority return to their respective communities with minimal postinjury support. This represents a critical missed opportunity for improving the delivery of comprehensive trauma care and wraparound services for firearm-injury survivors.

Disparities in trauma care are well-described, with significant research aimed at understanding differences in outcomes based on patient- and ecological-level determinants of health.^20,21,22^ Prior research has demonstrated that patients from economically disadvantaged communities are generally more vulnerable to injury, have more severe injuries, and suffer worse outcomes.^9,11^ Consistent with this work, we found that the largest proportion of patients in both injury cohorts presenting to trauma centers—a reflection of injury severity—were from the most distressed communities. We further found that patients from the most distressed communities had a significantly higher odds of presenting with a firearm injury, rather than an MVC injury, and that housing vacancy rate and poverty rate were the factors with the highest adjusted odds of presenting with firearm injury. Recent work has demonstrated similar associations between markers of community-level disadvantage and firearm injury in adults. In a single-city analysis, Dalve et al^10^ found significant association between neighborhood deprivation and firearm injuries, while Van Dyke et al^12^ reported county-level data demonstrating that higher proportions of patients presenting to the ED with firearm injuries were from low socioeconomic status communities. Additionally, similar to our findings, Harfouche et al^13^ reported on a single-center experience comparing patients injured with firearms with injuries by other mechanisms, and found that patients with firearm injuries lived in communities with higher rates of poverty, while Fornari et al^23^ found that patients injured by firearms are also at greater risk of reinjury than individuals of other injury types. With respect to the significance of housing vacancy rate and poverty in our findings, addressing factors in the built environment of distressed communities may be a target for local policymakers. For example, studies in Philadelphia have shown that efforts to rehabilitate vacant lots and facades of vacant houses have led to lower rates of firearm and other violence in those communities.^24,25,26^ Despite the consistency of these findings, these studies have been limited to single geographic areas or single institutions and have had small sample sizes. By contrast, this study reports on the experiences of 104 trauma centers from across the US, treating over 60 000 adults, 9507 of whom suffered a firearm injury.

Among the patients injured by firearms in this study, the majority were discharged with no rehabilitation services. More specifically, less than one-third of patients injured with firearms were referred for psychosocial care following treatment. Patients sustaining firearm injuries have unique needs throughout their posthospital recovery. A 2016 qualitative analysis of self-identified postdischarge needs among patients with firearm injuries found that patients had challenges when returning for follow-up care, were worried about reentering their communities safely with new disabilities, and reported anxiety, fear, and flashbacks, but lacked access to mental health care.^27^ Moreover, several studies have reported on the higher incidence of acute stress disorder and post-traumatic stress disorder (PTSD) among firearm injury survivors in the year after injury, and found associations with patient-level demographic SDOH and uptake of postdischarge care.^28,29,30^ Herrera-Escobar et al^31^ found that over half of patients injured by firearms contacted at 6 to 12 months screened positive for PTSD, had daily pain, and had not returned to work, and that in comparison with those injured in MVCs, patients sustaining firearm injuries were 2 to 3 times more likely to have daily pain and screen positive for PTSD. This raises concern for the need for improved screening and referral guidelines for postdischarge care that account for the SDOH that may commonly follow from firearm injuries. A 2022 ACS COT survey of over 300 trauma centers found that while 99% of centers routinely screen for alcohol use, only 50% and 30% of centers screen for PTSD and firearm injury, respectively.^32^ Additionally, only one-third of responding centers reported having programs supporting violence prevention. While expansions of psychiatric care, social work engagement, case management, and wraparound services would be ideal, these are ultimately precious and limited resources. Using our study’s findings that patients seen at trauma centers for firearm injuries more frequently come from communities with high economic disadvantage, residential zip code and corresponding DCI could be used as embedded screening tools to direct postdischarge resources for patients who may have greatest needs.

### Limitations

There are several potential limitations in this work. First, no data were available for patients with MVC-related injuries discharged from the ED, necessitating excluding this population entirely. Limiting inclusion to hospitalized patients may bias the findings toward those patients residing in higher levels of community distress with fewer social supports allowing for prompt discharge. Second, the collection of postdischarge data was a part of the expanded inclusion criteria for the ACS COT Firearm Study, and were not available for patients with MVC injuries . Third, we used zip code to determine community distress, which is not as granular as census blocks and thus may miss more socially meaningful associations. However, data at the zip code level often underestimates associations^33,34^ and provides a general understanding of the patterns of area economic disadvantage. While we examined differences in DCI based on its inclusion of key measures of economic wellness and demonstrated similar performance to other measures of economic advantage such as the Area Deprivation Index and the Social Vulnerability Index, which are used by both researchers and government programs.^35,36^ Finally, there are likely differences in the proportion of firearm vs MVC injuries in a community that are treated at the regional trauma center. For example, in most communities with trauma centers, such as those included in this study, patients who are Black, male, and uninsured are more likely to be treated at trauma centers.^37^

## Conclusions

Among adults treated at trauma centers, patients from highly distressed communities were disproportionately affected by firearm-related injuries as opposed to MVC injuries. The built environment and poverty may be important upstream modifiable factors to reduce firearm injuries. At the patient level, two-thirds of patients treated for firearm injuries at trauma centers are discharged without psychosocial services, highlighting an opportunity to update screening guidelines and improve care specific to firearm injuries at trauma centers. Community-level measures of disadvantage may prove instrumental for allocating postdischarge care resources to patients with the greatest need.

## Notes

Supplement 1. **eTable 1.** Patient Demographics Comparing Adult Motor Vehicle Injury to Assault Firearm Injury Patients Presenting Alive to 104 Trauma Centers **eTable 2.** Clinical Characteristics by Injury Type **eTable 3.** Postdischarge Services Among Surviving Adult Patients With Assault Firearm Injuries by ISS **eTable 4.** Distressed Communities Index (DCI) of Patient Residential Zip Codes by Injury Type **eTable 5.** Adjusted Odds Ratios (OR) of Assault Firearm Injury vs MVC-Related Injury

Supplement 2.
Data Sharing Statement
