# Supplementary material for: Community-Level Disadvantage of Adults With Firearm- vs Motor Vehicle–Related Injuries
Source: JAMA Netw Open. 2024 Jul 5;7(7):e2419844. doi: 10.1001/jamanetworkopen.2024.19844 (PMC11227070; doi:10.1001/jamanetworkopen.2024.19844)
Supplement: Supplement 1. — eTable 1. Patient Demographics Comparing Adult Motor Vehicle Injury to Assault Firearm Injury Patients Presenting Alive to 104 Trauma Centers eTable 2. Clinical Characteristics by Injury Type eTable 3. Postdischarge Services Among Surviving Adult Patients With Assault Firearm Injuries by ISS eTable 4. Distressed Communities Index (DCI) of Patient Residential Zip Codes by Injury Type eTable 5. Adjusted Odds Ratios (OR) of Assault Firearm Injury vs MVC-Related Injury [file jamanetwopen-e2419844-s001.pdf]

Supplemental Online Content

Agoubi LL, Banks S, Hink AB, et al. Community-level disadvantage of adults with firearm vs motor vehicle related injuries. *JAMA Netw Open.* 2024;7(6):e2419844. doi:10.1001/jamanetworkopen.2024.19844

eTable 1. Patient Demographics Comparing Adult Motor Vehicle Injury to Assault Firearm Injury Patients Presenting Alive to 104 Trauma Centers

eTable 2. Clinical Characteristics by Injury Type

eTable 3. Postdischarge Services Among Surviving Adult Patients With Assault Firearm Injuries by ISS

eTable 4. Distressed Communities Index (DCI) of Patient Residential Zip Codes by Injury Type

eTable 5. Adjusted Odds Ratios (OR) of Assault Firearm Injury vs MVC-Related Injury

This supplemental material has been provided by the authors to give readers additional information about their work.

© 2024 Agoubi LL et al. *JAMA Network Open.*

Supplemental Table 1. Patient demographics comparing adult motor vehicle injury to assault firearm injury patients presenting alive to 104 trauma centers.

	Firearm Injury (Assault only) (N=6753)	Motor Vehicle Collision Injury (N=53474)	Overall (N=60227)
**Age**			
Mean (SD)	32.6 (11.3)	44.5 (18.0)	43.2 (17.8)
Median [IQR]	30 [24, 39]	41 [29, 58]	39 [28, 57]
19-30	3485 (51.6%)	15509 (29.0%)	18994 (31.5%)
31-40	1889 (28.0%)	10391 (19.4%)	12280 (20.4%)
41-50	839 (12.4%)	7854 (14.7%)	8693 (14.4%)
51-64	448 (6.6%)	10926 (20.4%)	11374 (18.9%)
65+	92 (1.4%)	8794 (16.4%)	8886 (14.8%)
**Sex**			
Female	859 (12.7%)	18150 (33.9%)	19009 (31.6%)
Male	5595 (82.9%)	34496 (64.5%)	40091 (66.6%)
Non-Binary	0 (0%)	8 (0.0%)	8 (0.0%)
Missing	299 (4.4%)	820 (1.5%)	1119 (1.9%)
**Race**			
White	1514 (22.4%)	33663 (63.0%)	35177 (58.4%)
Black	4338 (64.2%)	12251 (22.9%)	16589 (27.5%)
Other	531 (7.9%)	4644 (8.7%)	5175 (8.6%)
Asian	63 (0.9%)	986 (1.8%)	1049 (1.7%)
American Indian	42 (0.6%)	363 (0.7%)	405 (0.7%)
Pacific Islander	31 (0.5%)	228 (0.4%)	259 (0.4%)
>1 Race	12 (0.2%)	115 (0.2%)	127 (0.2%)
Missing	222 (3.3%)	1224 (2.3%)	1446 (2.4%)
**Ethnicity**			

© 2024 Agoubi LL et al. *JAMA Network Open*.

	Firearm Injury (Assault only) (N=6753)	Motor Vehicle Collision Injury (N=53474)	Overall (N=60227)
Not Hispanic or Latino	5358 (79.3%)	43888 (82.1%)	49246 (81.8%)
Hispanic or Latino	977 (14.5%)	7744 (14.5%)	8721 (14.5%)
Missing	418 (6.2%)	1842 (3.4%)	2260 (3.8%)
**Primary Method of Payment**			
Private/Commercial Insurance	1167 (17.3%)	23835 (44.6%)	25002 (41.5%)
Medicare	200 (3.0%)	6368 (11.9%)	6568 (10.9%)
Medicaid/Other Government	3231 (47.8%)	12689 (23.7%)	15920 (26.4%)
Self-Pay/Not Billed/Other	1800 (26.7%)	8621 (16.1%)	10421 (17.3%)
Missing	355 (5.3%)	1961 (3.7%)	2316 (3.8%)
**Facility Region**			
Midwest	2006 (29.7%)	15308 (28.6%)	17314 (28.7%)
Northeast	1106 (16.4%)	6602 (12.3%)	7708 (12.8%)
South	2117 (31.3%)	21309 (39.8%)	23426 (38.9%)
West	1524 (22.6%)	10255 (19.2%)	11779 (19.6%)

© 2024 Agoubi LL et al. *JAMA Network Open.*

Supplemental Table 2. Clinical characteristics by injury type.

	Firearm Injury (Assault Only) (N=6753)	Motor Vehicle Collision Injury (N=53474)	Overall (N=60227)
**Mortality**			
Deceased	863 (12.8%)	3161 (5.9%)	4024 (6.7%)
Survived	5890 (87.2%)	50313 (94.1%)	56203 (93.3%)
**Transport Mode**			
Air	477 (7.1%)	8006 (15.0%)	8483 (14.1%)
Ground Ambulance	5084 (75.3%)	43154 (80.7%)	48238 (80.1%)
Other	4 (0.1%)	18 (0.0%)	22 (0.0%)
Police	206 (3.1%)	71 (0.1%)	277 (0.5%)
Private/Public Vehicle/ Walk-in	966 (14.3%)	2099 (3.9%)	3065 (5.1%)
Missing	16 (0.2%)	126 (0.2%)	142 (0.2%)
**ED Discharge Disposition**			
Died in ED	541 (8.0%)	1101 (2.1%)	1642 (2.7%)
Floor/Observation Unit	2193 (32.5%)	25124 (47.0%)	27317 (45.4%)
ICU/SDU	1201 (17.8%)	18293 (34.2%)	19494 (32.4%)
OR	2768 (41.0%)	8145 (15.2%)	10913 (18.1%)
Missing	50 (0.7%)	811 (1.5%)	861 (1.4%)
**Total Length of Stay**			
Mean (SD)	7.50 (9.60)	8.29 (11.2)	8.20 (11.0)
Median [Min, Max]	4.00 [1.00, 132]	5.00 [1.00, 368]	5.00 [1.00, 368]
Missing	148 (2.2%)	109 (0.2%)	257 (0.4%)
**ICU Length of Stay**			

© 2024 Agoubi LL et al. *JAMA Network Open*.

	Firearm Injury (Assault Only) (N=6753)	Motor Vehicle Collision Injury (N=53474)	Overall (N=60227)
Mean (SD)	6.35 (8.56)	6.94 (8.57)	6.87 (8.57)
Median [Min, Max]	4.00 [1.00, 142]	4.00 [1.00, 138]	4.00 [1.00, 142]
Missing	4302 (63.7%)	35056 (65.6%)	39358 (65.3%)
**Hospital Discharge Disposition**	N=6,212	N=52,373	N=58,585
Died	322 (5.2%)	2060 (3.9%)	2382 (4.1%)
Home	4741 (76.3%)	37195 (71.0%)	41936 (71.6%)
Other	491 (7.9%)	1689 (3.2%)	2180 (3.7%)
SNF or other long-term care	611 (9.8%)	11035 (21.1%)	11646 (19.9%)
Transferred to acute care	28 (0.5%)	394 (0.8%)	422 (0.7%)
Missing	19 (0.3%)	0 (0%)	19 (0%)
**Alcohol Screen Result**			
Negative	3237 (47.9%)	24690 (46.2%)	27927 (46.4%)
Positive	1496 (22.2%)	11451 (21.4%)	12947 (21.5%)
Missing/Not Tested	2020 (29.9%)	17333 (32.4%)	1937 (32.1%)

© 2024 Agoubi LL et al. *JAMA Network Open*.

Supplemental Table 3. Post-discharge services among surviving adult patients with assault firearm injuries by ISS.

	<16 (N=4354)	16-24 (N=835)	25+ (N=691)	Overall (N=5890)
**Rehabilitation/Post-Discharge Needs**				
Any	698 (16.0%)	221 (26.5%)	325 (47.0%)	1246 (21.2%)
Inpatient sub-acute rehabilitation	218 (5.0%)	111 (13.3%)	235 (34.0%)	565 (9.6%)
Outpatient physical therapy	394 (9.0%)	88 (10.5%)	74 (10.7%)	557 (9.5%)
Outpatient occupational therapy	194 (4.5%)	56 (6.7%)	45 (6.5%)	295 (5.0%)
Outpatient speech therapy	13 (0.3%)	6 (0.7%)	12 (1.7%)	31 (0.5%)
Outpatient rehabilitation medicine	53 (1.2%)	10 (1.2%)	22 (3.2%)	85 (1.4%)
Missing	209 (4.8%)	43 (5.1%)	30 (4.3%)	283 (4.8%)
**Home Health Needs**				
Any	624 (14.3%)	168 (20.1%)	174 (25.2%)	969 (16.5%)
Nursing	252 (5.8%)	89 (10.7%)	90 (13.0%)	431 (7.3%)
Wound Care	287 (6.6%)	61 (7.3%)	60 (8.7%)	411 (7.0%)
Infusion therapy	3 (0.1%)	4 (0.5%)	3 (0.4%)	10 (0.2%)
Rehabilitation therapies	267 (6.1%)	72 (8.6%)	79 (11.4%)	418 (7.1%)
Missing	227 (5.2%)	69 (8.3%)	86 (12.4%)	383 (6.5%)
**Psychosocial Ancillary Services**				
Any	1062 (24.4%)	254 (30.4%)	257 (37.2%)	1575 (26.7%)
Social work/case manager	402 (9.2%)	92 (11.0%)	83 (12.0%)	578 (9.8%)
Child protective services	6 (0.1%)	2 (0.2%)	2 (0.3%)	10 (0.2%)
Psychologist	119 (2.7%)	32 (3.8%)	45 (6.5%)	196 (3.3%)

© 2024 Agoubi LL et al. *JAMA Network Open*.

	<16 (N=4354)	16-24 (N=835)	25+ (N=691)	Overall (N=5890)
Psychiatry	79 (1.8%)	31 (3.7%)	22 (3.2%)	132 (2.2%)
Hospital/community-based violence intervention programs	644 (14.8%)	137 (16.4%)	163 (23.6%)	945 (16.0%)
Intimate partner violence services	20 (0.5%)	5 (0.6%)	4 (0.6%)	29 (0.5%)
Housing services (shelter, transitional housing)	94 (2.2%)	19 (2.3%)	10 (1.4%)	123 (2.1%)
Missing	222 (5.1%)	56 (6.7%)	58 (8.4%)	338 (5.7%)

© 2024 Agoubi LL et al. *JAMA Network Open*.

Supplemental Table 4. Distressed Communities Index (DCI) of patient residential zip codes by injury type.

	Firearm Injury (Assault only) (N=6753)	Motor Vehicle Collision Injury (N=53474)	Overall (N=60227)
**DCI Numeric Score**			
Mean (SD)	68.5 (26.3)	54.8 (29.0)	56.3 (29.0)
Median [IQR]	76.0 [56.5, 95.5]	58.0 [33.0, 83.0]	60.3 [35.25, 85.35]
Missing	277 (4.1%)	1697 (3.2%)	1974 (3.3%)
**DCI Quintiles**			
1 (least distressed)	463 (6.9%)	8727 (16.3%)	9190 (15.3%)
2	685 (10.1%)	8552 (16.0%)	9237 (15.3%)
3	911 (13.5%)	9555 (17.9%)	10466 (17.4%)
4	1515 (22.4%)	11549 (21.6%)	13064 (21.7%)
5 (most distressed)	2902 (43.0%)	13394 (25.0%)	16296 (27.1%)
Missing	277 (4.1%)	1697 (3.2%)	1974 (3.3%)
**DCI Subcomponents**			
No HS Diploma (%)			
Median [Min, Max]	14.6 [0, 61.4]	11.7 [0, 72.0]	12.1 [0, 72.0]
Poverty Rate			
Median [Min, Max]	19.7 [1.80, 63.8]	14.1 [0, 81.2]	14.6 [0, 81.2]
Adults not Working (%)			
Median [Min, Max]	24.9 [3.70, 82.1]	21.9 [0, 97.2]	22.2 [0, 97.2]
Housing Vacancy Rate			
Median [Min, Max]	9.40 [0, 53.4]	7.50 [0, 55.3]	7.70 [0, 55.3]
Median Income Ratio			
Median [Min, Max]	69.7 [21.7, 250]	87.8 [4.10, 317]	86.4 [4.10, 317]
Change in Employment (%)			
Median [Min, Max]	3.50 [-71.5, 480]	4.20 [-89.1, 2610]	4.10 [-89.1, 2610]
Change in Establishments			

© 2024 Agoubi LL et al. *JAMA Network Open*.

	Firearm Injury (Assault only) (N=6753)	Motor Vehicle Collision Injury (N=53474)	Overall (N=60227)
Median [Min, Max]	1.80 [-53.6, 100]	2.50 [-82.1, 400]	2.40 [-82.1, 400]
Missing	277 (4.1%)	1697 (3.2%)	1974 (3.3%)

© 2024 Agoubi LL et al. *JAMA Network Open*.

Supplemental Table 5. Adjusted odds ratios (OR) of assault firearm injury vs MVC-related injury [a]

	OR (95% CI)
**Age Group (years)**	
19-30	Ref
31-40	0.82 (0.76, 0.88)
41-50	0.53 (0.48, 0.58)
51-64	0.22 (0.19, 0.25)
65+	0.09 (0.07, 0.12)
**Sex**	
Female	0.31 (0.28, 0.33)
Male	Ref
**Race**	
American Indian	1.88 (1.30, 2.73)
Asian	1.85 (1.36, 2.51)
Black	5.83 (5.35, 6.37)
Other	1.44 (1.24, 1.66)
Pacific Islander	3.55 (2.24, 5.64)
White	Ref
>1 race	2.16 (1.08, 4.33)
**Ethnicity**	
Not Hispanic/Latino	Ref
Hispanic/Latino	1.54 (1.37, 1.73)
**Payer Type**	
Private/commercial insurance	Ref
Medicare	1.91 (1.57, 2.34)
Medicaid/other government	3.91 (3.58, 4.27)
Self-pay/not billed/other	3.04, 2.75, 3.36)
**DCI Quintile**	
1 (least distressed)	Ref
2	1.15 (0.99, 1.33)
3	1.14 (0.99, 1.31)
4	1.36 (1.19, 1.55)
5 (most distressed)	1.77 (1.56, 2.02)
**DCI sub-component analysis**	
No High School Diploma	0.98 (0.92, 1.03)
Poverty	1.19 (1.11, 1.28)
Adults not working	0.92 (0.87, 0.98)
Housing Vacancy rate	1.13 (1.04, 1.22)
Median Income ratio	0.97 (0.95, 0.99)
Change in employment	0.99 (0.97, 1.01)
Change in establishments	1.01 (0.97, 1.05)

[a] *Clustering at the facility level was controlled for with a random intercept. ORs presented for the DCI sub-component analysis control for socio-demographic characteristics.*

© 2024 Agoubi LL et al. *JAMA Network Open.*
