# Supplementary material for: Community-Level Disadvantage of Adults With Firearm- vs Motor Vehicle–Related Injuries
Source: JAMA Netw Open. 2024 Jul 5;7(7):e2419844. doi: 10.1001/jamanetworkopen.2024.19844 (PMC11227070; doi:10.1001/jamanetworkopen.2024.19844)
Supplement: Supplement 2. — Data Sharing Statement [file jamanetwopen-e2419844-s002.pdf]

Data Sharing Statement

Agoubi. Community-Level Disadvantage of Adults with Firearm- vs Motor Vehicle–Related Injuries. *JAMA Netw Open*. Published July 05, 2024.
doi:10.1001/jamanetworkopen.2024.19844

Data
Data available: No

Additional Information
Explanation for why data not available: Data will be shred upon reasonable request
